# Conductive catalysis by subsurface transition metals

**DOI:** 10.1093/nsr/nwae015

**Published:** 2024-01-11

**Authors:** Xin Deng, Caiyan Zheng, Yangsheng Li, Zeyu Zhou, Jiamin Wang, Yihua Ran, Zhenpeng Hu, Fan Yang, Landong Li

**Affiliations:** Frontiers Science Center for New Organic Matter, College of Chemistry, Nankai University, Tianjin 300071, China; School of Physics, Nankai University, Tianjin 300071, China; School of Physical Science and Technology, ShanghaiTech University, Shanghai 201210, China; School of Physical Science and Technology, ShanghaiTech University, Shanghai 201210, China; School of Materials Science and Engineering, Nankai University, Tianjin 300350, China; School of Physical Science and Technology, ShanghaiTech University, Shanghai 201210, China; School of Physics, Nankai University, Tianjin 300071, China; School of Physical Science and Technology, ShanghaiTech University, Shanghai 201210, China; Frontiers Science Center for New Organic Matter, College of Chemistry, Nankai University, Tianjin 300071, China; School of Materials Science and Engineering, Nankai University, Tianjin 300350, China; Haihe Laboratory of Sustainable Chemical Transformations, Tianjin 300192, China

**Keywords:** electronic interaction, conductive catalysis, subsurface, transition metal

## Abstract

The nature of catalysis has been hotly pursued for over a century, and current research is focused on understanding active centers and their electronic structures. Herein, the concept of conductive catalysis is proposed and verified by theoretical simulations and experimental observations. Metallic systems containing buried catalytically active transitional metals and exposed catalytically inert main group metals are constructed, and the electronic interaction between them *via* metallic bonding is disclosed. Through the electronic interaction, the catalytic properties of subsurface transitional metals (Pd or Rh) can be transferred to outermost main group metals (Al or Mg) for several important transformations like semi-hydrogenation, Suzuki-coupling and hydroformylation. The catalytic force is conductive, in analogy with the magnetic force and electrostatic force. The traditional definition of active centers is challenged by the concept of conductive catalysis and the electronic nature of catalysis is more easily understood. It might provide new opportunities for shielding traditional active centers against poisoning or leaching and allow for precise regulation of their catalytic properties by the conductive layer.

## Teaser

Metallic systems containing buried catalytically active transitional metals and exposed catalytically inert main group metals show remarkable performance in several important reactions, providing evidence on conductive catalysis through electronic interaction via metallic bonding.

## INTRODUCTION

Heterogeneous catalysis plays a key role in the modern chemical industry and makes a significant contribution to the progress of human society. The knowledge of heterogeneous catalysis is developing, from the early chemisorption [1] to the active center [2], and then to the transition state [3,4] and the electronic structure [5]. The concept of active center or active site is straightforward, which can be simply catalogued as the Brønsted acid site, the Lewis acid site, the base site, the redox site, and the metal site [6]. Various strategies like alloying [7], doping [8], confining [9], and size regulating [10] have been executed to active sites to promote their catalytic properties. These strategies can be essentially understood as the electronic modulation of the active centers and the surroundings like the *d*-band-center and Fermi level [11,12]. A typical example is a ‘volcano plot’, where the reaction can be efficiently catalyzed only when the electronic interaction between the adsorbate and the substrate is neither too strong nor too weak [13–16]. Additives like alkali metals can stabilize transitional metal centers through electronic interaction and thus boost their catalytic properties owing to the huge electron donation effect [17–19]. Originally, the electronic effect leads to the redistribution of charge density, i.e. electron delocalization and transfer, between the active sites and the adjacent microenvironment. Taking the advantages of charge transfer between the active metal host and the inert metal substrate, the Cu-based single atom alloys are reported to exhibit good performance in reactions like alkyne semi-hydrogenation and methane activation [20–22]. On the other hand, the concept of an electron as a catalyst, proposed several decades ago [23,24], has been verified in multiple radical cascades including Heck-type reactions, cross-dehydrogenative coupling and so on [25]. Recently, the electron-accelerated molecular recognition process by catalytic amount of chemical extraneous electron source has been achieved, offering a strong paradigm for electron relevant catalysis [26]. In general, the electron media, including the electronic structure of active site and the surroundings as well as the electron delocalization and transfer are deeply involved in the catalytic process and may dominate the catalytic properties [27]. Understanding the electronic effect is essential to the nature of catalysis.

However, the electrons are ubiquitous to distinguish and the dynamic process is difficult to trace [28], veiling the electronic effect in catalysis. It has recently been disclosed that the metallic water solution can be obtained via electron donation from alkali metals [29], demonstrating the electron-mediated transfer of the metallic nature. We assume that catalysis might be transferred with electron or electron interaction as media. To verify the hypothesis, we designed a unique metal system consisting of exposed catalytically inert main group metals and buried catalytically active transitional metals. The electron transfer between the buried transitional metals and exposed main group metals in a single crystal model system can be visualized by scanning tunneling microscopy (STM) and verified by density functional theory (DFT) simulation. With the electronic interaction as media, the single crystal model systems Pd/Al and Rh/Al are predicted to be active in acetylene semi-hydrogenation and propylene hydroformylation, respectively. The concept of conductive catalysis is therefore taking shape, which provides new insight into the nature of catalysis and offers a new strategy toward robust industrial catalysts.

## RESULTS AND DISCUSSION

**Figure 1.**
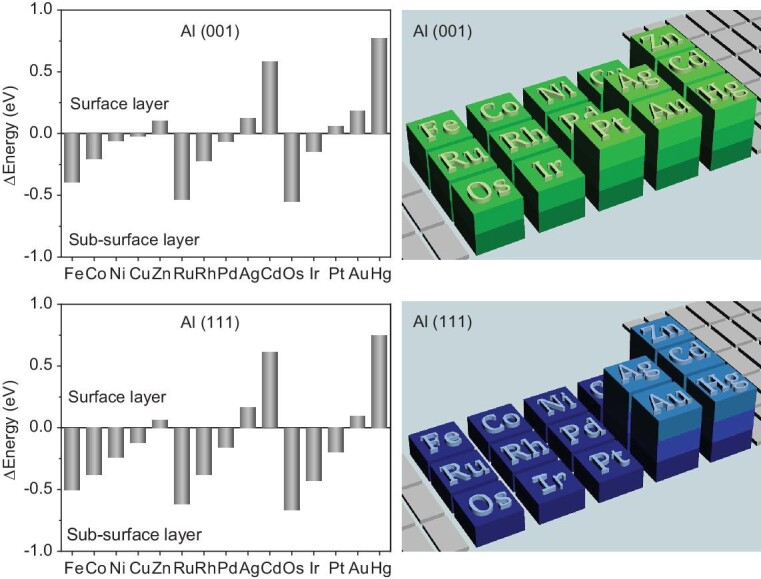
Location of *d*-block metal atoms in single crystal aluminum. Energy difference of *d*-block metal distribution in Al(001) and Al(111) from DFT calculations, ∆*E*_001_ = *E*_2L_ − *E*_1L_; ∆*E*_111_ = *E*_3L_ − *E*_1L_. Schematic illustration of *d*-block metal distribution in Al(001) and Al(111).

Aluminum, a main group metal, is the most abundant metal element in the Earth's crust and has relatively high electric conductivity (Table S1). It is generally inert in catalysis unless being doped or combined with a second transitional metal [30]. Herein, single crystal aluminum was selected as the inert substrate, and a series of *d*-block metals including Fe, Co, Ni, Cu, Zn, Ru, Rh, Pd, Ag, Cd, Os, Ir, Pt, Au and Hg were introduced to Al(001) and Al(111). DFT predictions show that some *d*-block metals exhibit a spontaneous sinking tendency and they can be well stabilized in the subsurface region of single crystal aluminum (Fig. 1 and Figs S1, S2). This can be rationalized by multiple factors including atomic radius, surface energy and deposition energy (Figs S3, S4 and Table S2). The sinking tendency of some *d*-block metals can also be observed over Mg(001) and Mg(100), sharing the analogous regulations (Figs S5–S9 and Table S2). In the specific case of Al(001), the *d*-block element shows sinking behavior when the atomic radius is smaller than 1.39 Å and the surface energy is larger than 0.05 eV/Å^2^. In principle, Pd and Rh atoms prefer to situate in the subsurface of both Al(001) and Al(111) while Ag atoms prefer to locate at the outer-surface (Fig. 1 and Table S2), constructing model systems with two distinct configurations. The Bader charge and the differential charge density analyses reveal that the electrons are enriched in the subsurface isolated Pd and Rh atoms, while the adjacent Al atoms appear to be in the electron-deficient state (Table S3). In other words, some of the valence electrons in Al atoms will transfer to Pd or Rh atoms *via* metallic bonding [31], driven by electrostatic attraction from the nucleus of Pd or Rh. The changes in the electronic structure of both Pd (Rh) and Al will probably bring about unconventional catalytic properties.

**Figure 2.**
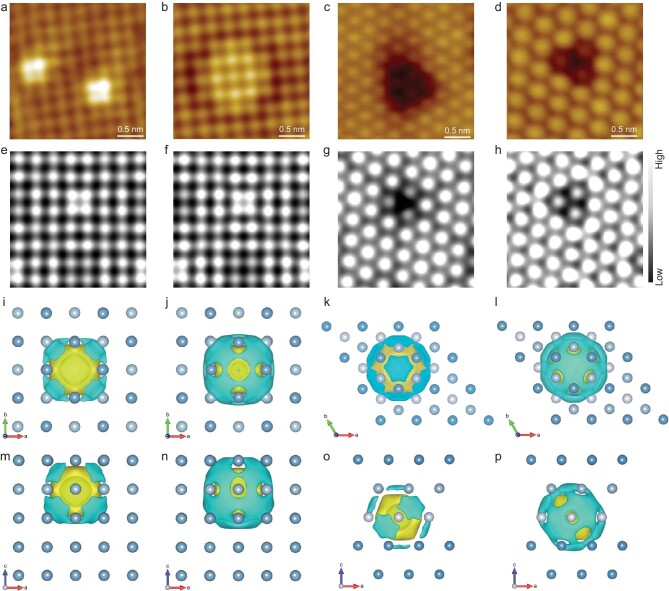
M/Al single crystal systems and their electronic states. (a–d) STM images of Pd/Al(001), Rh/Al(001), Pd/Al(111) and Rh/Al(111) after annealing to 600 K. (e–h) DFT-simulated STM images of an isolated metal atom in the subsurface region of Al(001) and Al(111), respectively, denoted as Pd/Al(001)-2 L, Rh/Al(001)-2 L, Pd/Al(111)-3 L and Rh/Al(111)-3 L. (i–p) Differential charge density of Pd/Al(001)-2 L, Rh/Al(001)-2 L, Pd/Al(111)-3 L and Rh/Al(111)-3 L structures from top (i–l) and side (m–p) views, respectively. Yellow and cyan isosurfaces indicate charge accumulation and depletion, respectively (isosurfaces = 0.0015 e/bohr^3^).

To confirm the predicted inter-diffusion tendency and visualize the electron transfer, Pd and Rh nanolayers were deposited on Al(001) and Al(111), respectively, and annealed at elevated temperatures with their surface structures monitored *in situ* with atomic-resolution STM. Pd and Rh atoms evaporated in ultra-high vacuum (UHV) to form nanoislands on Al(001) and Al(111) at 300 K. Upon annealing, both Pd and Rh nanoislands disappeared gradually at elevated temperatures and completely at >600 K on Al(001) and Al(111). Due to the larger surface energy of M/Al(001), the rates of alloying and inter-diffusion for Pd (Rh) on Al(001) are higher than those on Al(111) (Figs S10–S12). Accompanying the inter-diffusion of Pd and Rh atoms, the Pd/Al(001), Pd/Al(111), Rh/Al(001) and Rh/Al(111) surfaces turned flat after annealing to >600 K. Figure 2 shows further at the atomic level that the above annealed surfaces appear homogeneous with an Al skin (Fig. S13), while Pd and Rh atoms are located at the subsurface, forming subsurface-single-atom catalysts. STM image contrast of subsurface-single-atom Pd or Rh catalyst (Fig. 2) is drastically different from those surface alloys of Pd-Al and Rh-Al systems, as observed during the growth of Pd and Rh on Al single crystals (Figs S14, S15). Note that, Pd and Rh atoms at the subsurface of Al(001) caused typically bright contrast on the neighboring surface Al atoms, whereas Pd and Rh atoms at the subsurface of Al(111) induced dark contrast on the neighboring surface Al atoms (Fig. 2). DFT calculations and STM simulations suggest that Pd and Rh atoms are located in the Al(001) subsurface (Fig. 2a, b, e, f, and Figs S16, S17), whereas Pd and Rh atoms are located in the third layer of Al(111) (Fig. 2c, d, g, h, and Figs S18, S19), demonstrating the unique facet dependent behaviors. DFT calculations indicate that the charge transfer mechanism at different Al atomic layers results in different differential charge density (Fig. 2i–p), thereby showing varying atomic contrast in STM images. These results imply that the electron transfer mechanism determines the atomic structures of subsurface alloy of M/Al binary systems and can be used to tune the activity of the catalyst under realistic reaction conditions.

**Figure 3.**
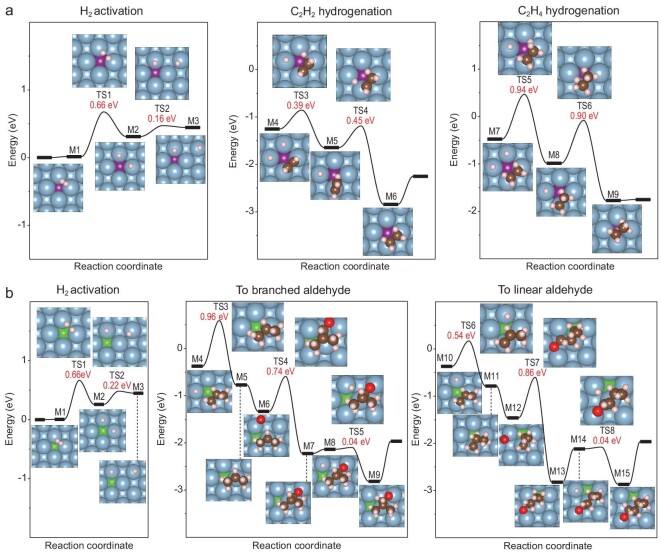
Catalysis by subsurface metal atoms in Al(001) through electron transfer. (a) Calculated reaction pathways and energy profiles of acetylene selective hydrogenation over Pd/Al(001) with atomic structures shown as insets. (b) Calculated reaction pathways and energy profiles of propylene hydroformylation over Rh/Al(001) with atomic structures shown as insets. Al, Pd, Rh, C, H and O atoms are shown in blue, purple, green, brown, white and red, respectively.

The reaction pathways of dihydrogen activation over Al or Mg substrate containing Pd and Rh in the outermost surface or subsurface region were explored. As shown in Figs S20–S22, the location of Pd or Rh in the substrate indeed shows some impacts on dihydrogen activation. Nevertheless, dihydrogen can be activated even if Pd or Rh are completely buried inside the subsurface region and inaccessible to dihydrogen molecules, hinting to non-contacting catalysis through electronic interaction. We then come to explore the catalytic properties of Pd/Al(001) and Rh/Al(001). Typically, the Pd/Al(001) model system can catalyze the semi-hydrogenation of acetylene to ethylene. The reaction follows the classical heterogeneous Horiuti–Polanyi mechanism with dihydrogen dissociation serving as the rate determining step (**TS1** = 0.66 eV) (Fig. 3a) [32]. The energy barrier of ethylene hydrogenation (**TS5*** =* 0.94 eV) is much higher than that of acetylene hydrogenation (**TS4*** =* 0.45 eV), ensuring high selectivity in semi-hydrogenation. The Rh/Al(001) model system can catalyze the hydroformylation of propylene (Fig. 3b). The reaction starts from dihydrogen activation (**TS1** = 0.66 eV) and overcomes the energy barrier of 0.96 eV (**TS3**) and 0.86 eV (**TS7**) to produce *iso*-butanal and *n*-butanal, respectively. Since the side reaction of propylene hydrogenation to byproduct propane bears distinctly higher energy barriers (Fig. S23; **TS4′** = 1.10 eV, **TS7′** = 0.97 eV), a high chemo-selectivity can be expected in propylene hydroformylation. The hydroformylation mechanism catalyzed by Rh/Al(001) is similar to that reported for Rh-based heterogeneous catalysts [33]. The semi-hydrogenation of acetylene and hydroformylation of propylene are also feasible over Pd/Al(111) and Rh/Al(111), respectively, as predicted by DFT calculations (Figs S24–S26). The catalytic reactions over M/Al(111) need to overcome a slightly higher energy barrier than that of M/Al(001). The aforementioned DFT calculations disclose the catalytic properties of Pd/Al and Rh/Al although Pd and Rh are completely buried inside an Al single crystal and inaccessible to reaction substrates. The surface Al atoms directly bonded to subsurface Pd or Rh serve as the apparent sites for reactant adsorption and activation, and the electronic structure of these surface Al sites are regulated by subsurface Pd or Rh *via* electronic interaction. The outermost Al atoms act as the conductive layer for catalysis. That is, the catalytic force, as initially proposed by Berzelius in 1936 [34], can be conductive like magnetic forces and electrostatic forces.

Guided by theoretical predictions and surface science observations, we can build real metallic systems with buried active centers for catalytic applications. Noble metals Pd and Rh are selected as active centers, and main group metals Al and Mg as inert substrate. The real metallic systems with different configurations were prepared and characterized by a series of spectroscopic techniques (Figs S27–S33). Briefly, high-sensitivity low-energy ion scattering spectroscopy depth profiles of Pd/Al and Rh/Al (Fig. S33) display no Pd or Rh signals on the outermost layer while these signals can be clearly observed in the subsurface region (2nd–7th layer), confirming that Pd and Rh species are completely buried inside the subsurface region of Al as predicted by DFT calculations (Fig. 1). The aberration-corrected scanning transmission electron microscopy images in Fig. S29 further demonstrate the atomically dispersed Pd and Rh species in the subsurface region of Al substrate, in good consistency with STM observations (Fig. 2). As expected, the Pd/Al catalyst exhibits good performance in the semi-hydrogenation of acetylene (87% ethylene selectivity at full acetylene conversion with an apparent activation energy of 71 kJ/mol) (Fig. S34a, b) as well as prominent stability without obvious declines in acetylene conversion or ethylene selectivity for >90 h (Fig. S34c). The dissociation of dihydrogen is determined to be the rate determining step with an H-D kinetic isotopic effect value of 2.9 (340 K) (Fig. S34d) and the reaction follows the classical Horiuti-Polanyi mechanism (Fig. S34e, f), in line with DFT calculations (Fig. 3a). Similarly, Pd/Mg exhibits good performance in acetylene semi-hydrogenation at distinctly lower reaction temperatures (Fig. S35). Besides, the Pd/Al catalyst can accommodate the semi-hydrogenation of a series of alkynes and alkynols in the liquid phase, overwhelming the industrial Lindlar catalyst (Table S4). Pd is known as the unique active component for Suzuki-coupling reaction and Pd/Al indeed exhibits remarkable performance in Suzuki-coupling reaction between bromobenzene and phenylboronic acid with an apparent activation barrier of 64 kJ/mol (Fig. S36a–c). Notably, Pd/Al offers a very high site-specific rate of 25 935 ${\mathrm{mo}}{{\mathrm{l}}}_{{\mathrm{C}} - {\mathrm{C}}}/{\mathrm{mo}}{{\mathrm{l}}}_{{\mathrm{Pd}}}/{\mathrm{h}}$ in this reaction at 323 K (Fig. S36d and Table S5) and shows good compatibility among various substrates of aryl halides and arylboronic acids (esters) (Table S6). In contrast, Pd/Mg is completely inactive for the Suzuki-coupling reaction under identical reaction conditions, although it contains Pd in the subsurface (Table 1). Rh/Al with buried Rh species in Al substrate exhibits remarkable catalytic performance in 1-hexene hydroformylation with an apparent activation energy of 56 kJ/mol (Fig. S37a–c). It gives a state-of-the-art specific rate of 21 376 ${\mathrm{mo}}{{\mathrm{l}}}_{{\mathrm{CHO}}}/{\mathrm{mo}}{{\mathrm{l}}}_{{\mathrm{Rh}}}/{\mathrm{h}}$ in 1-hexene hydroformylation at 433 K (Fig. S37d and Table S7) and meanwhile shows good compatibility in a broad scope of alkenes including aromatic and long chain alkenes (Table S8). Like the case of Pd/Mg, Rh/Mg is completely inactive in the hydroformylation of 1-hexene. According to the catalytic results summarized in Table 1, it can be stated that catalysis by Pd and Rh is conductive in real metal catalyst systems. Although the metallic bonding is highly suitable for the conduction of catalysis *via* facile electronic interaction, the changes or attenuation in catalytic force will occur especially in real catalyst systems. The catalytic reaction is very sensitive to the electronic structure of active centers, for example in the case of Suzuki-coupling by Pd and hydroformylation by Rh, even tiny changes in the catalytic force can completely change the apparent catalytic properties, while for the not so sensitive reaction to the electronic structure of active centers like semi-hydrogenation, subtle changes in the catalytic properties are obtained. Besides Pd and Rh, non-noble metals like Ni and Cu in Al substrate have been investigated for catalysis. As expected, Ni/Al and Cu/Al catalysts with buried Ni and Cu metal sites show remarkable performance in the selective hydrogenation of carbon–carbon triple bonds (Fig. S38), demonstrating the universality of M/Al systems in conductive catalysis.

**Table 1:**
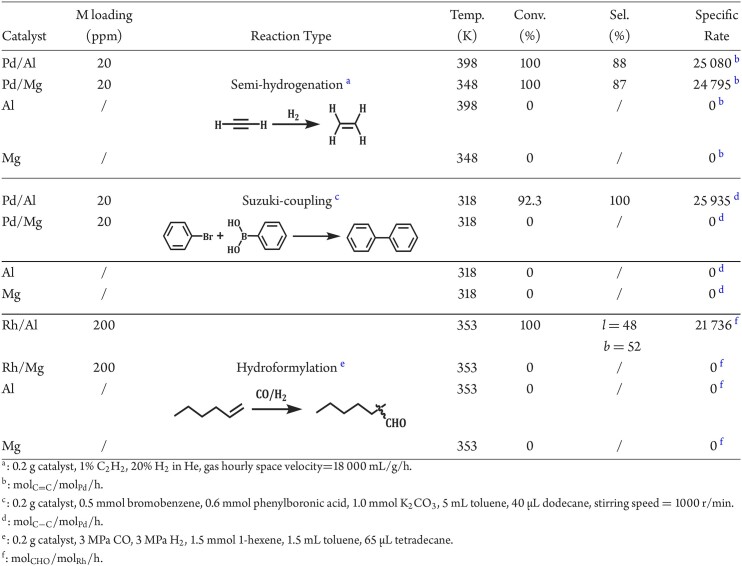
Catalytic properties of M/Al and M/Mg in several typical reactions.

## CONCLUSION

In summary, the sinking tendency of some *d*-block transitional metals in main group metal substrates (Al and Mg) has been proved theoretically and experimentally, constructing metallic systems with buried transitional metals in the subsurface region and facile electronic interaction *via* metallic bonding. Benefiting from the unique configurations of such metallic systems, we disclose that the catalytic properties of buried transitional metals can be transferred to the surface of catalytically inert main group metals and accordingly propose the concept of conductive catalysis. We expect that it will inspire reconsideration about the traditional definition of active centers and the electronic nature of catalysis. Robust metallic catalysts have been successfully developed for target reactions like semi-hydrogenation, Suzuki-coupling and hydroformylation with the active centers Pd or Rh in the traditional sense buried inside an Al or Mg substrate. This strategy can provide an efficient shield of the traditional active centers against poisoning or leaching by the conductive layer, in contrast to traditional nanoparticle systems [35]. More importantly, we suppose that the catalytic properties of buried transitional metals can be precisely regulated or completely altered when passing through the conductive layer.

## MATERIALS AND METHODS

Prior to use, commercial aluminum foam panels (YinFu Technology Co. Ltd) were pretreated with hot hydrochloric acid followed by aqua regia for 30 min, respectively. The processed aluminum foam was then washed with distilled water and dried in a vacuum at 333 K for 6 h. A series of metal precursors were dissolved in deionized water to acquire the aqueous solution of Pd(NO_3_)_2_, Rh(NO_3_)_2_, AgNO_3_, Cu(NO_3_)_2_, and Ni(NO_3_)_2_ with certain concentration. Then the dried aluminum foam was placed in the above solution and stirred for 3 h at 333 K under a flow of high-purity helium followed by washing with distilled water several times. The solid samples were dried in a vacuum at 333 K for 12 h, reduced in high-purity H_2_ at 573 K for 3 h, and annealed in helium at 673 K for 3 h to derive M/Al, labeled as Pd/Al, Rh/Al, Ag/Al, Cu/Al and Ni/Al, respectively.

All STM experiments were performed in a combined UHV system consisting of a low-temperature STM chamber (Crea Tec LT-STM, base pressure: <1.5 × 10^−10^ mbar), preparation chamber (base chamber: <1.5 × 10^−10^ mbar) and load-lock chamber (base pressure: <5 × 10^−8^ mbar).

The semi-hydrogenation of acetylene was conducted in a fixed-bed reactor under ambient pressure. The reactants and products were on-line analyzed by gel chromatography (GC, Techcomp DP7900) equipped with a flame ionization detector and a HP-PLOT/Q (Agilent, 30 m × 0.32 mm) capillary column using N_2_ as the carrier gas.

The Suzuki-coupling reaction was conducted in a 20-mL high-pressure autoclave equipped with a magnetic stirrer. The liquid products were collected by centrifugation and the products were then analyzed by GC (Techcomp DP7900) equipped with a flame ionization detector and a DB-1 (Agilent, 30 m × 0.32 mm) capillary column using N_2_ as the carrier gas.

The hydroformylation of alkenes was performed in a 20-mL high-pressure autoclave equipped with a magnetic stirrer. After the completion of the reaction, the liquid products were collected by centrifugation and analyzed by GC (Techcomp D7900P) equipped with a DB-1 (Agilent, 30 m × 0.25 mm) column as well as GC-mass spectrometry (Shimadzu GCMS-QP2010 SE) equipped with an RXI-5MS column (30 m, 0.25 mm i.d., stationary phase thickness of 0.25 μm).

## Supplementary Material

nwae015_Supplemental_File
