# Supplementary material for: Conductive catalysis by subsurface transition metals
Source: Natl Sci Rev. 2024 Jan 11;11(1):nwae015. doi: 10.1093/nsr/nwae015 (PMC10849361; doi:10.1093/nsr/nwae015)
Supplement: nwae015_Supplemental_File [file nwae015_supplemental_file.docx]

Supplementary Materials for

**Conductive catalysis by subsurface transition metals**

Xin Deng^1,†^, Caiyan Zheng^2,†^, Yangsheng Li^3,†^, Zeyu Zhou^3^, Jiamin Wang^4^,
Yihua Ran^3^, Zhenpeng Hu^2^*, Fan Yang^3^*, and Landong Li^1,4,5^*

^1^ Frontiers Science Center for New Organic Matter, College of Chemistry, Nankai University, Tianjin 300071, P.R. China

^2^ School of Physics, Nankai University, Tianjin 300071, P.R. China

^3^ School of Physical Science and Technology, ShanghaiTech University, Shanghai 201210, P.R. China

^4^ School of Materials Science and Engineering, Nankai University, Tianjin 300350, P.R. China

^5^ Haihe Laboratory of Sustainable Chemical Transformations, Tianjin 300192, P.R. China

† Equally contributed to this work.

* Corresponding authors Emails: [lild@nankai.edu.cn](mailto:lild@nankai.edu.cn) (L.L.); [zphu@nankai.edu.cn](mailto:zphu@nankai.edu.cn) (Z.H.); [fyang@shanghaitech.edu.cn](mailto:fyang@shanghaitech.edu.cn) (F.Y.)

**This PDF file includes:**

Figures S1 to S38

Tables S1 to S8

Supplementary References 36 to 72

**MATERIAL and METHOD**

**Chemical reagents.** Palladium nitrate (Pd(NO_3_)_2_), Rhodium nitrate (Rh(NO_3_)_3_), Silver nitrate (AgNO_3_), Copper nitrate Cu(NO_3_)_2_, Nickel nitrate Ni(NO_3_)_2_, Phenylacetylene (C_8_H_6_), 3-Chlorophenylacetylene (C_8_H_5_Cl), 2-Butyn-1-ol (C_4_H_6_O), 3-Methyl butynol (C_5_H_8_O), 4-Ethynyltoluene (C_9_H_8_), 1-Phenyl-1-propyne (C_9_H_8_), Di-phenylacetylene (C_14_H_10_), Dehydro-linalool (C_10_H_16_O), Bromobenzene (C_6_H_5_Br), Iodobenzene (C_6_H_5_I), 4-Bromotoluene (C_7_H_7_Br), 4-Iodotoluene (C_7_H_7_I), 4-Bromoanisole (C_7_H_7_BrO), 4-Iodoanisole (C_7_H_7_IO), 2-Bromotoluene (C_7_H_7_Br), 2-Iodotoluene (C_7_H_7_I), 3-Bromotoluene (C_7_H_7_Br), 3-Iodotoluene (C_7_H_7_I), Phenylboronic acid (C_6_H_7_BO_2_), 4-Tolylboronic acid (C_7_H_9_BO_2_), Phenylboronic acid pinacol ester (C_12_H_17_BO_2_), 4-Methoxyphenylboronic acid (C_7_H_9_BO_3_), 1-Hexene (C_6_H_12_), 1-Pentene (C_5_H_10_), 1-Octene (C_8_H_16_), 1-Decene (C_10_H_20_), 1-Undecene (C_11_H_22_), 1-Hexadecene (C_16_H_32_), Styrene (C_8_H_8_), 4-Chlorostyrene (C_8_H_7_Cl), 4-Methylstyrene (C_9_H_10_). All chemical reagents are of analytical grade and used without further treatment.

Acetylene (C_2_H_2_, 2% balanced with helium), hydrogen (H_2_, 20% balanced with helium), deuterium (D_2_, 100%), high purity hydrogen (H_2_, 100%), syngas (CO 50% and H_2_ 50%), helium (100%), ethylene (C_2_H_4_, 100%), propylene (C_3_H_6_, 100%) were supplied by Dalian Special Gas Co. Ltd.

**Synthesis of M/Al.** Prior to use, the commercial aluminum foam panels (YinFu Technology. CO. LTD) were pretreated with hot hydrochloric acid followed by aqua regia for 30 min, respectively. Then the processed aluminum foam was washed with distilled water and dried in vacuum at 333 K for 6 h. A series of metal precursors were dissolved in deionized water to acquire the aqueous solution of Pd(NO_3_)_2_, Rh(NO_3_)_2_, AgNO_3_, Cu(NO_3_)_2_ or Ni(NO_3_)_2_ with certain concentration. Then the dried aluminum foam was placed in the above solution, stirred for 3 h at 333 K under the flow of high purity helium followed by washing with distilled water for several times. The solid samples were dried in vacuum at 333 K for 12 h, reduced in high purity H_2_ at 573 K for 3 h, and annealed in helium at 673 K for 3 h to derive M/Al, labeled as Pd/Al, Rh/Al, Ag/Al, Cu/Al and Ni/Al, respectively.

**Synthesis of M/Al-1L.** The reference samples M/Al-1L, from which the metal species mainly exposed on the outermost surface of Al substrate, were prepared *via* similar processes but without the high temperature annealing step.

**Synthesis of M/Mg.** M/Mg samples were prepared *via* similar processes as those employed in M/Al, using commercial magnesium foil instead of aluminum foam as the substrate.

**Scanning electron microscopy (SEM).** The SEM images were taken on a JOL JSM-7800F microscope.

**Focused ion beam (FIB).** The FIB experiments were performed with Zeiss Crossbeam340 equipped with Ga source.

**Transmission electron microscopy (TEM)**. The TEM images and energy dispersive spectroscopy (EDS) element mapping were taken on a JEOL JEM-2800 microscope.

**Aberration corrected scanning transmission electron microscopy (AC-STEM).** The AC-STEM observations were performed using a probe aberration corrected microscopy JEOL JEM-ARM200F microscope.

**High-sensitivity low-energy ion scattering (HS-LEIS)**. The HS-LEIS spectra were recorded using an Ion-TOF Qtac100 instrument (*36*). To minimize the damage to the surface, helium and neon were selected as the ion source with a kinetic energy of 3 keV, an ion flux of 6000 pAm^-2^ and a spot size of 2 mm*2 mm.

**Time-of-flight secondary ion mass spectroscopy (TOF-SIMS).** The TOF-SIMS analyses were conducted on a TOF-SIMS 5-100 instrument (ION-TOF-GmbH), connected with a UHV preparation chamber. A sputter-ion source of O_2_^+^ with ion energy at 500 eV, a beam current of 10 nA, and raster size of 300 µm was used for depth profiles. The analysis was conducted with a Bi^+^ primary gun with a beam energy of 15 kV, a beam current of 0.6 pA, and a raster size of 50 µm.

**Fourier transform infrared spectra of CO adsorption (CO-FTIR).** The FTIR spectra of CO adsorption were collected on a Bruker INVENIO R spectrometer equipped with a liquid nitrogen cooled LN-MCT Mid detector with a resolution of 4 cm^-1^. The chamber equipped with a liquid nitrogen cooling system and a vacuum pump, which can accommodate a pressure range of 1000 Pa to 1 atm and the sample temperatures of 113 to 873 K. The catalyst was pretreated with 20% H_2_/He at 573 K for 1 h and cooled down to 298 K in helium. The background spectrum was taken at 298 K in flowing helium. Afterward, the sample was saturated with 1% CO/He and purged with helium at the same temperature for 1 h, and the corresponding FTIR spectra were recorded.

**Semi-hydrogenation of acetylene.** The semi-hydrogenation of acetylene over Pd/Al and Ni/Al catalysts were conducted in a fixed-bed reactor under ambient pressure. Typically, a gas mixture of 1% C_2_H_2_, 20% H_2_ balanced with He (flow rate = 60 mL/min, GHSV = 18000 mL/g/h) was introduced to reactor containing 0.2 g catalyst and the reaction was performed with ascending temperatures. The reactants and products were on-line analyzed by GC (Techcomp DP7900) equipped with a flame ionization detector and a HP-PLOT/Q (Agilent, 30 m × 0.32 mm) capillary column using N_2_ as the carrier gas.

Acetylene conversion and ethylene selectivity were calculated as followed:

$$Acetylene Conversion \%=\frac{[C_{2}H_{2}]_{\mathrm{inlet}}-[C_{2}H_{2}]_{\mathrm{outlet}}}{[C_{2}H_{2}]_{\mathrm{inlet}}}\times100\%$$

$$Ethylene Selectivity \%=\frac{[C_{2}H_{4}]_{\mathrm{outlet}}}{[C_{2}H_{2}]_{\mathrm{inlet}}}\times100\%$$

Kinetic analyses were also performed in the fixed-bed rector mentioned above and the reaction was conducted at low temperatures to keep the reaction in the kinetic regime (acetylene conversion <15%).

**Temperature-programmed desorption (TPD) studies.** The TPD experiments were performed on a fixed-bed reactor equipped with a downstream gas sampling mass spectrometer (Pfeiffer Omnistar). Typically, *ca.* 0.2 g catalyst was pretreated in the flowing He in the quartz tube at 573 K for 1 h. After cooling down to 303 K, the catalyst was saturated with C_2_H_2_, C_2_H_4_ or H_2_ and purged by He at 303 K for 1 h. Finally, the C_2_H_2_/C_2_H_4_/H_2_-TPD profiles were recorded in the flowing He at a heating rate of 10 K/min from 303 to 800 K.

**H_2_-D_2_-C_2_H_2_ pulse-response experiments.** The H_2_-D_2_-C_2_H_2_ pulse-response experiments were performed over Pd/Al catalyst in a fixed-bed reactor equipped with a downstream gas sampling mass spectrometer (Pfeiffer Omnistar). In a typical experiment, 0.2 g catalyst was pretreated with H_2_ at 573 K for 1 h. Then, a gas mixture containing 10% H_2_ and 10% D_2_ balanced with N_2_ was introduced to the reactor at the temperature of 403 K. Then the acetylene pulses (5 mL/min, 5 s) were fed into the reactor and the signal responses from mass spectrometer were recorded.

**Semi-hydrogenation of alkynes/alkynols in the liquid-phase**. The semi-hydrogenation of alkynes/alkynols in the liquid-phase were performed in a high-pressure autoclave with a magnetic stirrer. In a typical reaction, 0.2 g catalyst, 1 mmol alkynes/alkynols, 40 µL tetradecane as an internal standard and 5 mL ethanol (toluene) as solvent were added into the autoclave. The autoclave was purged with N_2_ several times to remove the residual air. Then the autoclave was charged with H_2_ and rapidly heated to the designated temperature at a stirring rate of 1000 rpm, which is adequate to exclude the external mass transfer limitation. After the completion of the reaction, the liquid products were collected by centrifugation and the products were then analyzed by GC (Techcomp DP7900) equipped with a flame ionization detector and a DB-1 (Agilent, 30 m × 0.25 mm) capillary column using N_2_ as the carrier gas.

**Suzuki-coupling reactions.** The Suzuki-coupling reaction was conducted in a 20 mL high-pressure autoclave equipped with a magnetic stirrer. In a typical reaction, 0.5 g catalyst, 0.5 mmol aryl halide, 0.6 mmol aryl boronic acid, 1 mmol potassium carbonate, 40 µl dodecane as an internal standard and 5 mL toluene as solvent were added into the autoclave and purged with He several times to remove the residual air. Then the autoclave was rapidly heated to 343 K at a stirring rate of 1000 rpm. After the completion of the reaction, the liquid products were collected by centrifugation and the products were then analyzed by GC (Techcomp DP7900) equipped with a flame ionization detector and a DB-1 (Agilent, 30 m × 0.32 mm) capillary column using N_2_ as the carrier gas.

**Hydroformylation of alkenes.** The hydroformylation of alkenes was performed in a 20 mL high-pressure autoclave equipped with a magnetic stirrer. In a typical reaction, 0.2 g catalyst, 1.5 mmol alkene, 65 µL tetradecane as an internal standard and 1.5 mL toluene as solvent were added into the autoclave and purged with He several times. Then the autoclave was charged with 3 MPa H_2_ and 3 MPa CO and rapidly heated to the designated temperature at a stirring rate of 1000 rpm, which is adequate to exclude the external mass transfer limitation. After the completion of the reaction, the liquid products were collected by centrifugation and analyzed by gas chromatography (Techcomp D7900P) equipped with a DB-1 (Agilent, 30 m × 0.25 mm) column as well as gas chromatography-mass spectrometry (Shimadzu GCMS-QP2010 SE) equipped with an RXI-5MS column (30 m, 0.25 mm i.d., stationary phase thickness of 0.25 µm).

**Single crystal system and scanning tunneling microscopy (STM).** All STM experiments were performed in a combined ultrahigh vacuum equipment (UHV) system which consisting of a low-temperature STM chamber (Crea Tec LT-STM, base pressure: <1.5*10^-10^ mbar), preparation chamber (base chamber: < 1.5*10^-10^ mbar) and load-lock chamber (base pressure: <5*10^-8^ mbar). The preparation chamber is equipped with an ion source of sputter cleaning (Prevac), mass spectroscopy (Hiden), leak-valve and electron beam evaporator (Focus). STM experiments are conducted at 78 K and used a mechanically cut Pt-Ir tip. The Al(001) and Al(111) are cleaned by cycles of Ar^+^ sputtering and UHV annealing to 800 K for 10 min. Clean Al(001) and Al(111) surfaces are checked by STM without any impurities (fig. S12). All STM images were processed with SPIP software (Image Metrology of Denmark).

**Computational details.** All spin-polarized density functional theory (DFT) calculations were implemented in the Vienna Ab-initio Simulation Package (VASP) package (*37,38*), with the projector augmented wave (PAW) pseudopotential (*39*) and the Perdew-Burke-Ernzerhof (PBE) (*40*) exchange-correlation functional of the generalized gradient approximation (GGA). A plane wave kinetic energy cut-off of 400 eV was used. The convergence criteria of force on each atom and total energy during structural optimization were set to 0.05 eV/Å and 10^-5^ eV, respectively. A 15 Å vacuum layer perpendicular to the surface was adopted to avoid interactions due to periodicity. For the Brillouin zone sampling, a 2×2×1 gamma-centred k-point mesh was used for the *p*(4√2×4√2) unit cell of Al(001), *p*(5×5) unit cell of Mg(001) surface and *p*(5×3) unit cell of Mg(100) surface, and a 3×3×1 gamma-centred k-point mesh was used for *p*(5×5) unit cell of Al(111) surface. The catalytic models with five atom layers were constructed, and the bottom two layers were fixed to keep bulk lattice positions while the other atom layers and adsorbates were allowed to relax. A *p*(1×1) unit cell of eight atom layers was modeled to calculate the surface energy ($\sigma$) of each *d*-block transition metal atom, where the top and the bottom two layers were relaxed until the convergence 1tolerance of force was below 0.02 eV/Å. The surface energy can be obtained by Equation (S1),

$\sigma=\frac{1}{2S}(E_{\mathrm{surface}}-N\times E_{\mathrm{atom}}^{\mathrm{bulk}})$	(S1)

where $E_{\mathrm{surface}}$ is the total energy of each metal surface, $E_{\mathrm{atom}}^{\mathrm{bulk}}$ is the energy per atom in the bulk phase, *N* is the number of atoms in the surface, and *S* is the area of each surface. The climbing image nudged-elastic band (CI-NEB) (*41*) and dimer methods (*42,43*) were used to search for transition states to determine reaction energy barrier and favorable reaction path.

# **Figure S1**. Total energy of *d*-block elements (Fe, Co, Ni, Cu, Zn, Ru, Rh, Pd, Ag, Cd, Os, Ir, Pt, Au, Hg) over Al(001). The most stable configurations are highlighted in blue with the corresponding motifs shown as insets.

# **Figure S2**. Total energy of *d*-block elements (Fe, Co, Ni, Cu, Zn, Ru, Rh, Pd, Ag, Cd, Os, Ir, Pt, Au, Hg) over Al(111). The most stable configurations are highlighted in blue with the corresponding motifs shown as insets.

# **Figure S3**. The correlation between atom radius, surface energy and deposition energy in M/Al(001) system.

# **Figure S4**. The correlation between atom radius, surface energy and deposition energy in M/Al(111) system.

# **Figure S5**. Location of *d*-block metal atoms in single crystal magnesium. Energy difference of *d*-block metal distribution in Mg(001) and Mg(100) from DFT calculations, ∆*E*_001_=*E*_3L/2L_-*E*_1L_; ∆*E*_100_=*E*_3L/2L_-*E*_1L_. Schematic illustration of *d*-block metal distribution in Mg(001) and Mg(100).

# **Figure S6**. Total energy of *d*-block elements (Fe, Co, Ni, Cu, Zn, Ru, Rh, Pd, Ag, Cd, Os, Ir, Pt, Au, Hg) over Mg(001). The most stable configurations are highlighted in blue with the corresponding motifs shown as insets.

# **Figure S7**. Total energy of *d*-block elements (Fe, Co, Ni, Cu, Zn, Ru, Rh, Pd, Ag, Cd, Os, Ir, Pt, Au, Hg) over Mg(100). The most stable configurations are highlighted in blue with the corresponding motifs shown as insets.

# **Figure S8**. The correlation between atom radius, surface energy and deposition energy in M/Mg(001) system.

# **Figure S9**. The correlation between atom radius, surface energy and deposition energy in M/Mg(100) system.

# **Figure S10**. The annealing kinetics of Pd/Al(001) and Pd/Al(111). Sample was annealed at designated temperatures and STM images were taken to track the subsidence process. Scale bar= 10 nm.

# **Figure S11**. The annealing kinetics of Rh/Al(001) and Rh/Al(111). Sample was annealed at designated temperatures and STM images were taken to track the subsidence process. Scale bar= 10 nm.

# **Figure S12**. STM images of Al(001) and Al(111) with the corresponding DFT simulation images.

# **Figure S13**. STM images of Al skin formed on the Al surface after the Pd(Rh) nano-islands on Al(111)/(001) annealing in UHV at 673 K for Pd/Al(111) and at 600 K for Pd/Al(001), Rh/Al(111) and Rh/Al(001).

# **Figure S14**. STM images of Pd/Al(001) and Pd/Al(111) surface alloys which formed by annealing the Pd nano-islands at 400 K for Pd/Al(001) and 600 K for Pd/Al(111), respectively.

# **Figure S15**. STM images of Rh/Al(001) and Rh/Al(111) surface alloys which formed by annealing the Rh nano-islands at 600 K for Rh/Al(001) and Rh/Al(111), respectively.

# **Figure S16**. STM simulation images of Pd/Al(001)-2L under different voltage (-1.0 V~1.0 V).

# **Figure S17**. STM simulation images of Rh/Al(001)-2L under different voltage (-1.0 V~1.0 V).

# **Figure S18**. STM simulation images of Pd/Al(111)-3L under different voltage (-1.0 V~1.0 V).

# **Figure S19**. STM simulation images of Rh/Al(111)-3L under different voltage (-1.0 V~1.0 V).

# **Figure S20**. Reaction pathways of dihydrogen activation over various model slabs.

# **Figure S21**. Optimized structures of dihydrogen activation over various model slabs: M/Al(001)-1L (**a**), M/Al(001) (**b**), M/Al(111)-1L (**c**) and M/Al(111) (**d**). ***Al, M and O atoms are shown in blue, purple and white balls, respectively.***

# **Figure S22**. Optimized structures of dihydrogen activation over various model slabs: M/Mg(001)-1L (**a**), M/Mg(001) (**b**), M/Mg(100)-1L (**c**) and M/Mg(100) (**d**). ***Mg, M and O atoms are shown in grey, purple and white balls, respectively.***

# **Figure S23**. Calculated reaction pathways and energy profiles of propylene hydrogenation to propane over Rh/Al(001) with the optimized structures shown as insets. Al, Rh, C and H atoms are shown in blue, green, brown and white, respectively.

# **Figure S24**. Calculated reaction pathways and energy profiles of acetylene selective hydrogenation over Pd/Al(111) with the optimized structures shown as insets. Al, Pd, C and H atoms are shown in blue, purple, brown and white, respectively.

# **Figure S25**. Calculated reaction pathways and energy profiles of propylene hydroformylation reaction over Rh/Al(111) with the optimized structures shown as insets. Al, Rh, C, H and O atoms are shown in blue, green, brown, white and red, respectively.

# **Figure S26**. Calculated reaction pathways and energy profiles of propylene hydrogenation to propane over Rh/Al(111) with the optimized structures shown as insets. Al, Rh, C and H atoms are shown in blue, green, brown and white, respectively.

# **Figure S27**. SEM images of Pd/Al, Rh/Al, Pd/Al-1L and Rh/Al-1L.

# **Figure S28**. STEM images and element mappings of Pd/Al-1L and Rh/Al-1L.

# **Figure S29**. AC-STEM images of Pd/Al and Rh/Al. Scale bar: 1 nm.

**Note:** AC-STEM images were taken over a specific layer poised perpendicularly to the surface of Pd/Al and Rh/Al samples, which was meticulously extracted through FIB.

# **Figure S30.** XPS of M/Al and M/Al-1L.

Note: XPS results confirm the metallic state of M in both M/Al-1L and M/Al samples. It should be mentioned that the Pd and Rh species in Pd/Al and Rh/Al exhibit slightly lower binding energies in comparison with Pd/Al-1L and Rh/Al-1L, indicating the stronger electron transfer from Al towards Pd or Rh.

# **Figure S31**. FTIR spectra of CO adsorption (10 min) and He purging (10 min) on Pd/Al, Pd/Al-1L, Rh/Al and Rh/Al-1L at 298 K.

**Note:** No CO vibrational features were observed on Pd/Al at 298 K after helium purging. However, Pd/Al-1L showed several peaks at 2065, 1955 and 1905 cm^-1^, attributing to linearly-bonded, bridge-bonded and triple-bonded CO on palladium clusters/ensembles, respectively (*44, 45*). Similarly, no adsorption signal could be observed on Rh/Al after helium purging. In contrast, for Rh/Al-1L, peaks at 2085 and 2010 cm^-1^ associated with the symmetric and asymmetric vibrations of gem-dicarbonyl doublet CO on single Rh atoms together with weak peaks attributed to linearly-bonded CO (2060 cm^-1^) and bridged-bonded CO (1860 cm^-1^) on Rh^0^ clusters (*46*) were observed.

# **Figure S32**. TOF-SIMS analyses of Pd/Al, Rh/Al and Ag/Al catalysts.

**Note**: The TOF-SIMS analyses confirm that Pd or Rh species are buried in Al or Mg substrates whereas Ag species mainly exposed on the outer-surface, in accordance with DFT calculations and the STM observations.

# **Figure S33**. HS-LEIS spectra of Pd/Al and Rh/Al samples at different etching depths with 4He^+^ or 20Ne^+^, respectively.

# **Figure S34**. **Catalytic performance and mechanism of acetylene hydrogenation over Pd/Al catalyst.** (**a**) Temperature-dependent behaviors of acetylene hydrogenation over Pd/Al catalyst; (**b**) Arrhenius plot for acetylene conversion over Pd/Al catalyst; Reaction conditions: 0.2 g catalyst, 1 % C_2_H_2_, 20 % H_2_ in He, GHSV=18000 mL/g/h; (**c**) Stability test of Pd/Al catalyst at 370 K. Reaction condition: 0.2 g catalyst, 1% C_2_H_2_, 20% H_2_, balanced with He, GHSV=18000 mL/g/h; (**d**) Primary isotope effect observed over Pd/Al catalyst in acetylene selective hydrogenation; (**e**) TPD profiles of H_2_, C_2_H_2_ and C_2_H_4_ over Pd/Al; (**f**) Pulse-response experiments of acetylene feeding to Pd/Al catalyst in H_2_-D_2_ stream at 360 K. Reaction condition: catalyst=0.2 g, 10 mL/min H_2_, 10 mL/min D_2_, 50 mL/min N_2_, with two acetylene pulse introduced intermittently.

# **Figure S35**. **Catalytic performance and mechanism of acetylene hydrogenation over Pd/Mg catalyst.** (**a**) Temperature-dependent behaviors of acetylene hydrogenation over Pd/Mg catalyst; (**b**) Arrhenius plot for acetylene conversion over Pd/Mg catalyst; Reaction conditions: 0.2 g catalyst, 1 % C_2_H_2_, 20 % H_2_ in He, GHSV=18000 mL/g/h; (**c**) Stability test of Pd/Mg catalyst at 370 K. Reaction condition: 0.2 g catalyst, 1% C_2_H_2_, 20% H_2_, balanced with He, GHSV=18000 mL/g/h; (**d**) Primary isotope effect observed over Pd/Mg catalyst in acetylene selective hydrogenation; (**e**) TPD profiles of H_2_, C_2_H_2_ and C_2_H_4_ over Pd/Mg; (**e**) TPD profiles of H_2_, C_2_H_2_ and C_2_H_4_ over Pd/Mg; (**f**) Pulse-response experiments of acetylene feeding to Pd/Mg catalyst in H_2_-D_2_ stream at 360 K. Reaction condition: catalyst=0.2 g, 10 mL/min H_2_, 10 mL/min D_2_, 50 mL/min N_2_, with two acetylene pulse introduced intermittently.

# **Figure S36**. **Catalytic performance of Suzuki-coupling reaction over Pd/Al catalyst.** (**a**) Time-dependent behaviors of Pd/Al catalyst in Suzuki-coupling reaction. Reaction conditions: 0.2 g catalyst, 0.5 mmol bromobenzene, 0.6 mmol phenylboronic acid, 1 mmol K_2_CO_3_, 5 mL toluene, 40 µL dodecane, temperature = 318 K, stirring speed= 1000 rpm; (**b**) Reaction rates of Suzuki-coupling reaction over Pd/Al catalyst. Reaction conditions: 0.2 g catalyst, 0.5 mmol bromobenzene, 0.6 mmol phenylboronic acid, 1 mmol K_2_CO_3_, 5 mL toluene, 40 µL dodecane, temperature = 308-323 K, stirring speed= 1000 rpm; (**c**) Arrhenius plot for Suzuki-coupling reaction over Pd/Al catalyst; (**d**) Comparison of various catalysts in Suzuki-coupling reaction in terms of specific rate and reaction temperature.

# **Figure S37**. **Catalytic performance of hydroformylation reaction over Rh/Al catalyst.** (**a**) Time-dependent behaviors of 1-hexene hydroformylation over Rh/Al catalyst under different temperatures. Reaction conditions: 0.2 g catalyst, 3 MPa CO, 3 MPa H_2_, 1.5 mmol 1-hexene, 1.5 mL toluene, 65 µL tetradecane, 353 K; (**b**) 1-Hexene hydroformylation rates over Rh/Al catalyst. Reaction conditions: 0.2 g catalyst, 3 MPa CO, 3 MPa H_2_, 1.5 mmol 1-hexene, 1.5 mL toluene, 65 µL tetradecane, temperature = 353-373 K; (**c**) Arrhenius plot for hydroformylation reaction over Rh/Al catalyst; (**d**) Comparison of various catalysts in linear α-olefin hydroformylation in terms of specific rate and reaction temperature.

# **Figure S38**. **Selective hydrogenation over Ni/Al and Cu/Al catalysts.** (**a**) Temperature-dependent behaviors of acetylene hydrogenation over Ni/Al catalyst; (**b**) Arrhenius plot for acetylene conversion over Ni/Al catalyst; Reaction conditions: 0.2 g catalyst, 1 % C_2_H_2_, 20 % H_2_ in He, GHSV=18000 mL/g/h; (**c**) Selective hydrogenation of phenyl-acetylene, 3-chlorophenylacetylene, 4-ethynyltoluene and 1-phenylpropyne over Cu/Al catalyst; Reaction conditions: 0.1 g catalyst, 0.5 mmol alkyne substrates, 5 ml toluene, 40 µl dodecane, 2 MPa H_2_, temperature= 423-453 K, time= 8-12 h, stirring speed= 1000 rpm.

# **Table S1**. The conductivity and crustal abundance of element *^(47)^*.

| Element | Conductivity (10^6^/cm/Ω) | Crustal abundance (ppm) |
| --- | --- | --- |
| Ag | 0.63 | 0.07 |
| Cu | 0.596 | 50 |
| Au | 0.452 | 0.0011 |
| Al | 0.377 | 82000 |
| Mg | 0.226 | 23000 |
| Ni | 0.143 | 80 |

# **Table S2**. The parameters of *d*-block elements and single crystal.

| *d*-block element | Atomic radius (Å) | Surface Energy (eV/Å^2^) ^[a]^ | | | ΔEnergy (eV) ^[b]^ | | | |
| --- | --- | --- | --- | --- | --- | --- | --- | --- |
|  |  | (001) | (111) | (100) | Al(001) | Al(111) | Mg(001) | Mg(100) |
| Fe | 1.24 | 0.201 | 0.172 | 0.201 | -0.393 | -0.508 | -0.252 | -0.219 |
| Co | 1.25 | 0.157 | 0.278 | 0.157 | -0.204 | -0.384 | -0.240 | -0.169 |
| Ni | 1.25 | 0.139 | 0.121 | 0.139 | -0.058 | -0.243 | -0.189 | -0.063 |
| Cu | 1.28 | 0.089 | 0.082 | 0.089 | -0.022 | -0.123 | -0.088 | 0.071 |
| Zn | 1.34 | 0.023 | 0.057 | 0.045 | 0.101 | 0.064 | -0.028 | 0.033 |
| Ru | 1.33 | 0.164 | 0.207 | 0.187 | -0.533 | -0.617 | -0.539 | -0.505 |
| Rh | 1.35 | 0.147 | 0.126 | 0.147 | -0.221 | -0.383 | -0.378 | -0.263 |
| Pd | 1.38 | 0.096 | 0.083 | 0.096 | -0.064 | -0.158 | -0.206 | -0.052 |
| Ag | 1.45 | 0.050 | 0.045 | 0.050 | 0.124 | 0.163 | -0.088 | 0.047 |
| Cd | 1.51 | 0.009 | 0.031 | 0.023 | 0.579 | 0.610 | 0.029 | 0.073 |
| Os | 1.34 | 0.186 | 0.243 | 0.214 | -0.550 | -0.666 | -0.606 | -0.623 |
| Ir | 1.36 | 0.179 | 0.142 | 0.179 | -0.149 | -0.429 | -0.425 | -0.348 |
| Pt | 1.39 | 0.116 | 0.092 | 0.116 | 0.058 | -0.200 | -0.224 | -0.081 |
| Au | 1.44 | 0.054 | 0.044 | 0.054 | 0.818 | 0.095 | -0.083 | 0.061 |
| Hg | 1.51 | 0.004 | 0.005 | 0.004 | 0.770 | 0.747 | 0.075 | 0.110 |
| ^[a]^: $\sigma=\frac{1}{2S}(E_{\mathrm{surface}}-N\times E_{\mathrm{atom}}^{\mathrm{bulk}})$ ^[b]^: Δ*E*= *E*_3L/2L_-*E*_1L_ | | | | | | | | |

# **Table S3**. The total Bader charge (e) on Al atoms located in the nearest (Al-1) and next nearest neighbor (Al-2) of the dopant atoms in the Pd (Rh)/Al structures.

| Atom | Pd/Al(001) | Rh/Al(001) | Pd/Al(111) | Rh/Al(111) |
| --- | --- | --- | --- | --- |
| Pd (Rh) | 1.725 | 2.377 | 1.646 | 2.095 |
| Al-1 (1L) | -0.291 | -0.349 | -0.109 | -0.105 |
| Al-2 (1L) | -0.280 | -0.318 | -0.047 | -0.062 |
| Al-1 (2L) | -0.626 | -0.744 | -0.350 | -0.535 |
| Al-2 (2L) | -0.227 | -0.138 | 0.073 | 0.111 |
| Al-1 (3L) | -0.841 | -1.280 | -0.694 | -0.944 |
| Al-2 (3L) | 0.370 | 0.397 | -0.034 | -0.045 |

# **Table S4**. Selective hydrogenation of alkynes and alkynols over Pd/Al and Lindlar catalysts.

|  | | | Pd/Al (~20 ppm Pd) ^[a]^ | | | | Lindlar (~5% Pd) ^[b]^ | | |
| --- | --- | --- | --- | --- | --- | --- | --- | --- | --- |
| Entry | Substrate | Product | Conv.  (%) | Sel.  (%) | Rate ^[c]^ | Conv.  (%) | | Sel.  (%) | Rate ^[c]^ |
| 1 |  |  | 94.0 | 94.0 | 3917 | 95.0 | | 92.3 | 6.2 |
| 2 |  |  | 90.8 | 91.7 | 3691 | 93.2 | | 92.8 | 6.1 |
| 3 |  |  | 95.2 | 94.9 | 4004 | 90.3 | | 91.5 | 5.9 |
| 4 |  |  | 62.5 | 86.7 | 2402 | 98.9 | | 32.5 | 2.3 |
| 5 |  |  | 90.1 | 91.6 | 3658 | 100 | | 50.1 | 3.5 |
| 6 |  |  | 35.5 | 93.4 | 1470 | 100 | | 90.2 | 6.4 |
| 7 |  |  | 39.6 | 100 | 1755 | 100 | | 88.0 | 6.2 |
| 8 |  |  | 70.2 | 93.2 | 2900 | 100 | | 68.2 | 4.8 |
| ^[a]^: Reaction conditions: 0.2 g catalyst, 1 mmol substrate, 5 mL ethanol, 40 µL tetradecane, pressure= 0.6 MPa, time= 6 h, temperature = 333 K, stirring speed = 1000 rpm; ^[b]^: Reaction conditions: 0.05 g catalyst, 1 mmol substrate, 3 mL ethanol, 40 µL tetradecane, pressure= 0.6 MPa, time= 6 h, temperature = 333 K, stirring speed = 1000 rpm; ^[c]^: Specific rate of product formation in the unit of $\mathrm{mol}_{C=C}/\mathrm{mol}_{\mathrm{Pd}}/h$. | | | | | | | | | |

# **Table S5**. Comparison of Pd-based catalysts in Suzuki-coupling reaction

| Catalyst | Time (h) | T  (K) | Yield  (%) | Rate ^[b]^ | Substrates | Ref. |
| --- | --- | --- | --- | --- | --- | --- |
| MPOS-Pd | 0.008 | 353 | 50 | 7095 | Bromobenzene + Phenylboronic acid | *48* |
| Pd-ECN | 0.17 | 393 | 63 | 549 | Bromobenzene + Phenoborate pinnaol ester | *49* |
| Pd_1_/3DOM-CeO_2_ | 0.25 | 298 | 19 | 751 | Iodobenzene + Phenylboronic acid | *50* |
| Pd_1_/3DOM-CeO_2_ | 0.25 | 298 | 13 | 520 | Bromobenzene + Phenylboronic acid | *50* |
| Pd_1_/3DOM-CeO_2_ | 0.25 | 298 | 4 | 163 | Chlorobenzene + Phenylboronic acid | *50* |
| Pd@CMK-3 | 0.083 | 333 | 100 | 2800 | Bromobenzene + Phenylboronic acid | *51* |
| Pd@mZ-50 | 0.5 | 333 | 94 | 4050 | Iodobenzene + 4-Methoxyphenylboronic | *52* |
| Pd@PdO-graphene | 1 | 353 | 96 | 25 | 4-Nitrobromobenzene + Phenylboronic acid | *53* |
| Pd/Al_2_O_3_-CeO_2_ | 3 | 353 | 97 | 32 | Bromobenzene + Phenylboronic acid | *54* |
| Pd/Al_2_O_3_-CeO_2_ | 1 | 353 | 57 | 6843 | Bromobenzene + Phenylboronic acid | *54* |
| Pd_1_-Ti_0.87_O_2_ | 0.5 | 303 | 95.5 | 11110 | 4-Bromotoluene + Phenylboronic acid | *55* |
| Pd@EVOH | 2 | 408 | 100 | 530 | 4-Methoxyiodobenzene + Phenylboronic acid | *56* |
| GO-Pd | 0.16 | 353 | 27 | 39000 | 3-Bromobenzaldehyde +  4-Methoxyphenylboronic acid | *57* |
| Pd_1_/3DOM-CeO_2_ | 0.016 | 348 | 18 | 44527 | Bromobenzene + Phenylboronic acid | *50* |
| Pd/Al | 0.17 | 323 | 33 | 25935 | Bromobenzene + Phenylboronic acid | This work |
| ^[a]^: Specific rate of product formation in the unit of $\mathrm{mol}_{C-C}/\mathrm{mol}_{\mathrm{Pd}}/h$. | | | | | | |

# **Table S6**. Substrate scope of Suzuki-coupling reaction over Pd/Al catalyst.

|  | | | | |
| --- | --- | --- | --- | --- |
| Entry | Aryl halide | Phenylboronic acid | Yield (%) ^[a]^ | Rate ^[b]^ |
| 1 |  |  | 92.3 | 25935 |
| 2 |  |  | 91.6 | 18614 |
| 3 |  |  | 90.8 | 22001 |
| 4 |  |  | 89.5 | 25801 |
| 5 |  |  | 90.6 | 16334 |
| 6 |  |  | 95.6 | 16800 |
| 7 |  |  | 92.9 | 16961 |
| 8 |  |  | 88.9 | 19724 |
| 9 |  |  | 93.4 | 16358 |
| 10 |  |  | 92.5 | 21913 |
| 11 |  |  | 88.1 | 23378 |
| 12 |  |  | 90.9 | 24135 |
| 13 |  |  | 61.6 | 12517 |
| 14 |  |  | 77.2 | 16582 |
| 15 |  |  | 68.5 | 13059 |
| 16 |  |  | 71.6 | 15982 |
| ^[a]^: 0.2 g catalyst, 0.5 mmol aryl halide, 0.6 mmol phenylboronic acid, 1 mmol K_2_CO_3_, 5 mL toluene, 40 µL dodecane, temperature =323 K, time= 2 h; ^[b]^: Specific rate of production formation in the unite of $\mathrm{mol}_{C-C}/\mathrm{mol}_{\mathrm{Pd}}/h$. | | | | |

# **Table S7**. Comparison of Rh-based catalysts in linear α-olefin hydroformylation

| Catalysts | P (MPa) | T (K) | Time  (h) | Conv. (%) | Rate ^[a]^ | l/b | Substrate | Ref. |
| --- | --- | --- | --- | --- | --- | --- | --- | --- |
| [Co(acac)(DPPBz)](BF_4_) | 9 | 433 | 0.083 | 87.3 | 6192 | -- | 1-hexene | *58* |
| [rac-Rh_2_H_2_(µ-CO)_2_(CO)_4_(et,ph-P_4_)]^2+^ | 0.62 | 363 | / | / | 4380 | 33 | 1-hexene | *59* |
| Rh(PPh_3_)_3_ | 3 | 373 | 2 | 64 | 5000 | -- | propene | *60* |
| [Rh(cod)acac]PPh_3_ | 3 | 393 | 2 | 75 | 1255 | 1.5 | 1-octene | *61* |
| [Ru(CO)_4_]_n_/MWCNT | 5 | 423 | 5 | 95 | 0.9 | 2 | 1-hexene | *62* |
| HRh(CO)(PPh_3_)_3_ | 4 | 373 | 1 | 98.8 | 2455 | 2.57 | 1-hexene | *63* |
| Rh-Zn^II^-TPP | 2 | 298 | 24 | 2.5 | 29.1 | 2.4 | 1-octene | *64* |
| Rh(acca)(CO)_2_-(tropos,R,R) | 2 | 348 | 1 | 1 | 750 | 0.35 | propene | *65* |
| Rh/NAPHOS | 10 | 393 | 16 | 76 | 475 | 99 | 1-pentene | *66* |
| Rh/Biphephos | 3 | 433 | 4 | 88 | 8200 | 99 | 1-Hexene | *67* |
| Rh/ZnO-nw | 1.6 | 373 | 12 | 86 | 833 | / | 1-hexene | *46* |
| Rh/CoO | 3 | 373 | 2 | 64 | 2065 | / | propene | *68* |
| Rh/TPPTS | 4 | 353 | 4 | 97.4 | 392 | / | 1-octene | *69* |
| Rh_2_P@C | 4 | 373 | 5 | 100 | 17788 | 0.78 | 1-hexene | *70* |
| Rh@ZIF-8 | 5 | 373 | 12 | 92 | 76.7 | 0.9 | 1-hexene | *71* |
| RhZn/SBA-15 | 3 | 373 | 2 | 99.7 | 1341 | / | 1-hexene | *72* |
| Rh/Al | 6 | 433 | 0.083 | 46.753 | 21736 | 0.76 | 1-hexene | This work |
| ^[a]^: Specific rate of product formation in the unit of $\mathrm{mol}_{\mathrm{CHO}}/\mathrm{mol}_{\mathrm{Rh}}/h$. | | | | | | | | |

# **Table S8**. Substrate scope of alkene hydroformylation over Rh/Al catalyst ^[a].^

|  | | | | |
| --- | --- | --- | --- | --- |
| Entry | Substrate | Product | Yield (%) | |
|  |  |  | *b* | *l* |
| 1^[b]^ |  |  | 98 | |
| 2^[b]^ |  |  | 52 | 46 |
| 3 |  |  | 68 | 32 |
| 4 |  |  | 52 | 48 |
| 5 |  |  | 42 | 58 |
| 6 |  |  | 41 | 56 |
| 7 |  |  | 55 | 39 |
| 8 |  |  | 42 | 55 |
| 9 |  |  | 68 | 32 |
| 10 |  |  | 72 | 28 |
| 11 |  |  | 68 | 32 |
| ^[a]^: Reaction conditions; 0.2 g catalyst, 3 MPa CO, 3 MPa H_2_, 1.5 mmol substrate, 1.5 mL toluene, 65 µL tetradecane, temperature= 353 K, time= 10 h, stirring speed= 1000 rpm. | | | | |

# **Supplementary References**

36. Li Y, Hu J and Ma D *et al.* Disclosure of the surface composition of TiO_2_-supported gold-palladium bimetallic catalysts by high-sensitivity low-energy ion scattering spectroscopy. *ACS Catal.* 2018; **8**: 1790–5.
37. Kresse G and Hafner J. Ab initio molecular dynamics for liquid metals. *Phys. Rev. B* 1993; **47**: 558–61.
38. Kresse G and Furthmüller J. Efficiency of ab-initio total energy calculations for metals and semiconductors using a plane-wave basis set. *Comput. Mater. Sci.* 1996; **6**: 15–50.
39. Blöchl PE. Projector augmented-wave method. *Phys. Rev. B* 1994; **50**: 17953–79.
40. Perdew JP, Burke K and Ernzerhof M. Generalized gradient approximation made simple. *Phys. Rev. Lett.* 1996; **77**: 3865–8.
41. Henkelman G, Uberuaga BP and Jónsson H. Climbing image nudged elastic band method for finding saddle points and minimum energy paths. *J. Chem. Phys.* 2000; **113**: 9901–4.
42. Henkelman G and Jónsson H. A dimer method for finding saddle points on high dimensional potential surfaces using only first derivatives. *J. Chem. Phys.* 1999; **111**: 7010–22.
43. Kästner J and Sherwood P. Superlinearly converging dimer method for transition state search. *J. Chem. Phys.* 2008; **128**: 014106.
44. Rogers SM, Catlow CRA and Chan CE *et al.* Tandem site- and size-controlled Pd nanoparticles for the directed hydrogenation of furfural. *ACS Catal.* 2017; **7**: 2266–74.
45. Dong C, Gao Z and Li Y *et al.* Fully exposed palladium cluster catalysts enable hydrogen production from nitrogen heterocycles. *Nat. Catal.* 2022; **5**: 485–93.
46. Lang R, Li T and Matsumura D *et al.* Hydroformylation of olefins by a rhodium single-atom catalyst with activity comparable to RhCl(PPh_3_)_3_. *Angew. Chem. Int. Ed.* 2016; **55**: 16054–8.
47. Periodic Table of Elements. EnvironmentalChemistry.com. 1995-2023. https://EnvironmentalChemistry.com/yogi/periodic/ (21 September 2023, date last accessed).
48. Li B and Zeng HC. Minimalization of metallic Pd formation in Suzuki reaction with a solid-state organometallic catalyst. *ACS Appl. Mater. Interfaces* 2020; **12**: 33827–37.
49. Chen Z, Vorobyeva E and Mitchell S *et al.* A heterogeneous single-atom palladium catalyst surpassing homogeneous systems for Suzuki coupling. *Nat. Nanotechnol.* 2018; **13**: 702–7.
50. Tao X, Long R and Wu D *et al.* Anchoring positively charged Pd single atoms in ordered porous ceria to boost catalytic activity and stability in Suzuki coupling reactions. *Small* 2020; **16**: 2001782.
51. Wang Z, Chen W and Han Z *et al.* Pd embedded in porous carbon (Pd@CMK-3) as an active catalyst for Suzuki reactions: Accelerating mass transfer to enhance the reaction rate. *Nano Res.* 2014; **7**: 1254–62.
52. Ke W, Cui T and Yu Q *et al.* Mesoporous H-ZSM-5 nanocrystals with programmable number of acid sites as “solid ligands” to activate Pd nanoparticles for C–C coupling reactions. *Nano Res.* 2018; **11**: 874–81.
53. Jiang B, Song S and Wang J *et al.* Nitrogen-doped graphene supported Pd@PdO core-shell clusters for C-C coupling reactions. *Nano Res.* 2014; **7**: 1280–90.
54. Borkowski T, Dobosz J and Tylus W *et al.* Palladium supported on Al_2_O_3_-CeO_2_ modified with ionic liquids as a highly active catalyst of the Suzuki-Miyaura cross-coupling. *J. Catal.* 2014; **319**: 87–94.
55. Jin Y, Lu F and Yi D *et al.* Engineering electronic structure of single-atom Pd site on Ti_0.87_O_2_ nanosheet via charge transfer enables C-Br cleavage for room-temperature Suzuki coupling. *CCS Chem.* 2021; **3**: 1453–62.
56. Fernández E, Rivero-Crespo MA and Domínguez I *et al.* Base-controlled Heck, Suzuki, and Sonogashira reactions catalyzed by ligand-free platinum or palladium single atom and sub-nanometer clusters. *J. Am. Chem. Soc.* 2019; **141**: 1928–40.
57. Scheuermann GM, Rumi L and Steurer P *et al.* Palladium nanoparticles on graphite oxide and its functionalized graphene derivatives as highly active catalysts for the Suzuki-Miyaura coupling reaction. *J. Am. Chem. Soc.* 2009; **131**: 8262–70.
58. Hood DM, Johnson RA and Carpenter AE *et al.* Highly active cationic cobalt(II) hydroformylation catalysts. *Science* 2020; **367**: 542–8.
59. Aubry DA, Bridges NN and Ezell K *et al.* Polar phase hydroformylation: The dramatic effect of water on mono- and dirhodium catalysts. *J. Am. Chem. Soc.* 2003; **125**: 11180–1.
60. Wang L, Zhang B and Wang S *et al.* Atomic-level insights in optimizing reaction paths for hydroformylation reaction over Rh/CoO single-atom catalyst. *Nat. Commun.* 2016; **7**: 14036.
61. Piras I, Jennerjahn R and Jackstell R *et al.* A general and efficient iridium-catalyzed hydroformylation of olefins. *Angew. Chem. Int. Ed.* 2011; **50**: 280–4.
62. Oresmaa L, Moreno MA and Jakonen M *et al.* Catalytic activity of linear chain ruthenium carbonyl polymer [Ru(CO)_4_]_n_ in 1-hexene hydroformylation. *Appl. Catal. A Gen.* 2009; **353**: 113–6.
63. Mukhopadhyay K, and Chaudhari RV. Heterogenized HRh(CO)(PPh)_3_)_3_ on zeolite Y using phosphotungstic acid as tethering agent: A novel hydroformylation catalyst. *J. Catal.* 2003; **213**: 73–7.
64. Jongkind LJ, Elemans JAAW and Reek JNH. Cofactor controlled encapsulation of a rhodium hydroformylation catalyst. *Angew. Chem. Int. Ed.* 2019; **58**: 2696–9.
65. Iu L, Fuentes JA and Janka ME *et al.* High isoaldehyde selectivity in the hydroformylation of short-chain alkenes. *Angew. Chem. Int. Ed.* 2019; **58**: 2120–4.
66. Klein H, Jackstell R and Wiese KD *et al.* Highly selective catalyst systems for the hydroformylation of internal olefins to linear aldehydes. *Angew. Chem. Int. Ed.* 2001; **40**: 3408–11.
67. Vogl C, Paetzold E and Fischer C *et al.* Highly selective hydroformylation of internal and terminal olefins to terminal aldehydes using a Rhodium-BIPHEPHOS-catalyst system. *J. Mol. Catal. A Chem.* 2005; **232**: 41–4.
68. Amsler J, Sarma BB and Agostini G *et al.* Prospects of heterogeneous hydroformylation with supported single atom catalysts. *J. Am. Chem. Soc.* 2020; **142**: 5087–96.
69. Zhao Y, Zhang X and Sanjeevi J *et al.* Hydroformylation of 1-octene in Pickering emulsion constructed by amphiphilic mesoporous silica nanoparticles. *J. Catal.* 2016; **334**: 52–9.
70. Galdeano-Ruano C, Lopes CW and Meira MD *et al.* Rh_2_P nanoparticles stabilized by carbon patches for hydroformylation of olefins. *ACS Appl. Nano Mater.* 2021; **4**: 10743–53.
71. Hou C, Zhao G and Ji Y *et al.* Hydroformylation of alkenes over rhodium supported on the metal-organic framework ZIF-8. *Nano Res.* 2014; **7**: 1364–9.
72. Chen M, Gupta G and Ordonze CW *et al.* Intermetallic nanocatalyst for highly active heterogeneous hydroformylation. *J. Am. Chem. Soc.* 2021; **143**: 20907–15.
